# Transparent Al-Doped ZnO Thin Films for High-Sensitivity NO_2_ Gas Sensing

**DOI:** 10.3390/s25123622

**Published:** 2025-06-09

**Authors:** So-Young Bak, Se-Hyeong Lee, Hyeongrok Jang, Minseong Kim, Sungjae Kim, Moonsuk Yi

**Affiliations:** Department of Electrical and Electronics Engineering, Pusan National University, Busan 46241, Republic of Korea; bso6459027@pusan.ac.kr (S.-Y.B.); shlee19@pusan.ac.kr (S.-H.L.); rokmanv@pusan.ac.kr (H.J.); minseong8540@pusan.ac.kr (M.K.); keehab1208@pusan.ac.kr (S.K.)

**Keywords:** Al-doped ZnO, oxygen vacancies, gas sensor, NO_2_, Trimethylaluminum

## Abstract

**Highlights:**

**What are the main findings?**

- Al-doped ZnO thin films were deposited using atomic layer deposition, with trimethylaluminum inducing oxygen vacancies via its strong reducing nature.
- The sensor achieved 66.2× higher sensitivity and a 50 °C lower operating temperature.

**What is the implication of the main finding?**

- Controlled introduction of oxygen vacancies enabled low-temperature, high-sensitivity NO_2_ detection while maintaining high optical transparency.
- The sensor is suitable for transparent, low-power environmental monitoring systems.

**Abstract:**

This study developed a transparent NO_2_ gas sensor with enhanced sensing performance and high optical transmittance. Al-doped ZnO thin films were deposited by atomic layer deposition, which was chosen for its capability to precisely control surface chemistry at the atomic scale. Oxygen vacancies were effectively introduced by utilizing trimethylaluminum, a strongly reducing Al_2_O_3_ precursor, thereby increasing carrier concentration and enhancing gas-sensing performance. By adjusting the Al doping level, the optimized device achieved a 50 °C reduction in operating temperature, a 66.2-fold increase in sensitivity at 150 °C, and shortened response and recovery times. The morphology, crystallinity, and elemental distribution were analyzed using transmission electron microscopy, selected area electron diffraction, and energy-dispersive X-ray spectroscopy, while chemical bonding states were investigated via X-ray photoelectron spectroscopy. Optical properties were characterized using UV–visible spectroscopy, confirming an average transmittance of approximately 80% in the visible range. These results demonstrate the promise of transparent oxide gas sensors for integration into next-generation electronics and Internet of Things-based environmental monitoring systems.

## 1. Introduction

The advent of transparent electronic devices has introduced a paradigm shift, offering novel solutions to the limitations of conventional devices across various fields [1,2,3]. In the gas sensor domain, this advancement, coupled with the progress of Internet of Things (IoT) technologies, has enabled real-time environmental monitoring by integrating sensors into wearable devices such as smartwatches [4,5,6]. Furthermore, gas sensors have expanded their applications into medical fields, including disease diagnosis [7,8]. However, traditional opaque gas sensors face challenges in practical applications due to their restrictions on installation locations and aesthetic limitations [9,10]. To address these issues, transparent gas sensors have been proposed, offering seamless integration onto diverse surfaces such as windows and displays [11]. Benefiting from their excellent compatibility with existing electronic devices, transparent gas sensors are emerging as a promising technology for next-generation gas detection systems.

Among the various gases requiring monitoring, nitrogen dioxide (NO_2_) stands out as a critical pollutant due to its relevance in environmental monitoring, industrial safety, and especially in medical diagnostics, where it serves as a biomarker for respiratory diseases such as asthma and chronic obstructive pulmonary disease [12,13]. To meet the demands of these applications, sensors must be capable of detecting NO_2_ concentrations ranging from several parts per million (ppm) to sub-ppm or even parts per billion (ppb) [12,14,15]. This necessitates sensing platforms that offer high sensitivity and selectivity, along with long-term stability and low power consumption under practical operating conditions.

Metal oxide semiconductors (MOSs) with wide band gaps are transparent in the visible region and exhibit high gas response, establishing them as essential research materials for the development of transparent gas sensors [16,17,18]. ZnO, a representative n-type MOS, is widely utilized as a primary material for gas sensors due to its excellent compatibility with various fabrication techniques and high sensitivity to toxic gases [19,20]. To improve the performance of MOS-based gas sensors, various strategies such as heterojunction formation and catalyst incorporation have been explored [16,21]. However, these methods complicate fabrication processes and pose challenges to achieving device uniformity and reproducibility.

Atomic layer deposition (ALD) is a thin-film deposition technique based on self-limiting surface reactions, providing excellent conformality and chemical precision. These characteristics enable accurate control over dopant incorporation and surface composition, making ALD highly suitable for gas sensor fabrication [22,23]. Trimethylaluminum (TMA), a commonly used precursor in ALD for Al_2_O_3_ thin-film deposition, exhibits strong reducing properties. When deposited onto pre-existing oxide semiconductor films, such as TiO_2_ or ZnO, TMA induces the formation of oxygen vacancies [24,25,26]. Oxygen vacancies in n-type MOSs serve as electron donors by introducing shallow energy levels near the conduction band, thereby increasing electron density and enhancing electron mobility [27,28,29,30]. They also act as active sites for NO_2_ adsorption by providing localized electronic states that promote surface charge transfer [29,30]. This defect engineering approach serves as a foundation for significantly improving the sensitivity and response characteristics of gas sensors.

In this study, Al-doped ZnO (AZO) thin films were deposited using ALD, and the process was designed to enhance gas-sensing efficiency by promoting effective charge modulation through compositional and defect control. While Al doping is known to influence oxygen vacancy concentrations to some extent, conventional strategies generally do not provide direct control over defect formation [31,32,33]. In this study, we employ the reducing chemistry of TMA during ALD to intentionally induce oxygen vacancies alongside Al incorporation, enabling simultaneous compositional and defect engineering. This dual functionality eliminates the need for additional catalyst layers or heterostructures, which are often required to enhance sensing performance. As a result, our approach simplifies the fabrication process while maintaining high transparency and achieving improved NO_2_ sensitivity, faster response and recovery, and lower operating temperature. The device exhibited approximately 80% transmittance in the visible region, making it suitable for integration into transparent electronic systems. These results highlight its potential as a practical and innovative gas sensor technology for next-generation electronic devices.

## 2. Materials and Methods

**Figure 1:**
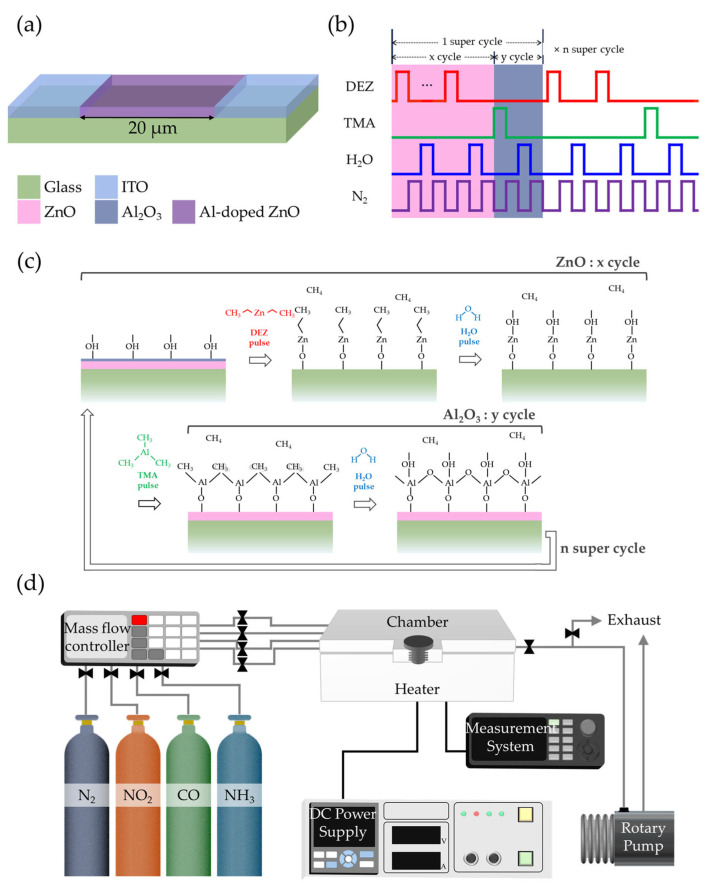
Schematic illustrations of (**a**) the fabricated gas sensor, (**b**) ALD pulse sequence for AZO deposition, (**c**) surface reaction mechanism during one super-cycle, and (**d**) gas-sensing measurement setup.

To fabricate transparent devices, glass substrates were utilized with indium tin oxide (ITO) as the electrode material. The ITO layer deposited on the glass substrate was patterned using an Nd:YAG laser to create a linear gap with a width of 20 µm. AZO served as the gas-sensing material, with the complete device structure illustrated in Figure 1a. The substrates underwent sequential ultrasonic cleaning in trichloroethylene, methanol, acetone, and deionized water (H_2_O) for 10 min each to remove organic residues and surface impurities. Subsequently, UV treatment was performed for 15 min to eliminate residual carbon-based organic contaminants and enhance surface reactivity for uniform precursor adsorption. The ALD process was carried out at a deposition temperature of 140 °C.

For AZO thin-film deposition, diethylzinc (DEZ) and TMA were used as precursors for ZnO and Al_2_O_3_, respectively, with H_2_O as the reactant. N_2_ gas served as both the carrier and purge gas. The pulsing sequence of precursors and purge gas in the ALD process is illustrated in Figure 1b, while Figure 1c shows a schematic of the surface reactions during deposition. Each ZnO deposition cycle consisted of DEZ exposure for 0.3 s, followed by N_2_ purge for 40 s, H_2_O exposure for 0.3 s, and another N_2_ purge for 40 s. Similarly, each Al_2_O_3_ deposition cycle comprised TMA exposure for 0.3 s, N_2_ purge for 20 s, H_2_O exposure for 0.3 s, and N_2_ purge for 20 s.

To investigate the effect of Al concentration on gas-sensing properties, the ratio of DEZ/H_2_O cycles (*x*) to TMA/H_2_O cycles (*y*) was varied as 100:0 (pristine ZnO), 50:1, 33:1, 25:1, 20:1, and 17:1. Since *y* remained constant at 1 for all samples except pristine ZnO, the AZO thin-film devices were designated according to their *x* values as AZO50, AZO33, AZO25, AZO20, and AZO17. Each super-cycle consisted of *x* cycles of DEZ/H_2_O followed by *y* cycles of TMA/H_2_O, and the number of super-cycles was adjusted such that the total number of ZnO cycles was approximately 100. All specimens were annealed at 600 °C for 1 h in a tube furnace after the deposition process, with the samples positioned at the center of the heating zone.

The morphological and crystalline properties of the AZO films were characterized using transmission electron microscopy (TEM, Talos F200X, Thermo Fisher Scientific, Waltham, MA, USA) equipped with selected area electron diffraction (SAED). Elemental composition was analyzed by energy-dispersive X-ray spectroscopy (EDS, XFlash 6T, Bruker, Billerica, MA, USA). In addition, surface morphology and complementary elemental analysis were performed using a field-emission scanning electron microscope (FE-SEM, JSM-7900F, JEOL, Tokyo, Japan) equipped with an EDS system (Ultim Extreme, Oxford Instruments, Abingdon, UK) operated at an acceleration voltage of 15 kV. X-ray photoelectron spectroscopy (XPS, AXIS SUPRA, Kratos Analytical, Manchester, UK) was employed to investigate the chemical composition and bonding states of the samples. XPS measurements were conducted using a monochromatic Al Kα X-ray source (1486.6 eV) under ultra-high vacuum conditions (<5 × 10^−10^ Torr) with an overall energy resolution of <0.48 eV. Optical properties were measured using UV–visible spectroscopy (UV–Vis, V-730, Jasco, Tokyo, Japan) in the wavelength range of 200–800 nm.

Gas-sensing measurements were conducted in a clean room environment with relative humidity maintained below 50%. As shown in Figure 1d, the sensing setup consisted of a closed test chamber connected to mass flow controllers for accurate control of gas composition and flow rate. Target gases NO_2_, carbon monoxide (CO), and ammonia (NH_3_) supplied at 100 ppm were diluted with N_2_ to achieve the desired concentrations. The total flow rate was fixed at 100 sccm during all measurements. After each exposure cycle, the chamber was purged with pure N_2_ at the same flow rate to restore the baseline condition. The gas response was defined as R_g_/R_a_ for oxidizing gases and R_a_/R_g_ for reducing gases, where R_a_ and R_g_ represent the resistance of the film in air and target gas, respectively. The response time was defined as the time required to reach 90% of the saturation state, while the recovery time was the time taken to return to 10% of the initial state.

## 3. Results and Discussion

### 3.1. Structural and Morphological Properties

**Figure 2:**
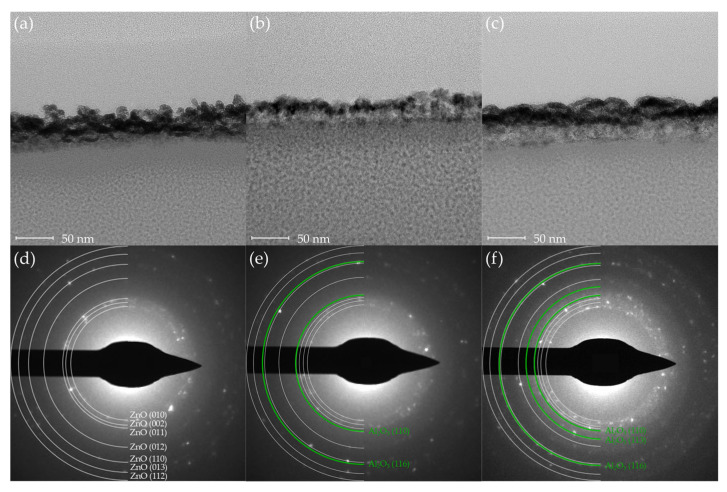
Cross-sectional TEM images of (**a**) ZnO, (**b**) AZO25, and (**c**) AZO17 thin films. Corresponding SAED patterns are shown in (**d**) ZnO, (**e**) AZO25, (**f**) AZO17.

To examine the thickness of the thin films, TEM samples were prepared using a focused ion beam system (FIB, Scios, Thermo Fisher Scientific, Waltham, MA, USA). Figure 2 presents cross-sectional TEM images and corresponding SAED patterns of the ZnO, AZO25 and AZO17 specimens. The measured film thickness was significantly greater than the expected value based on the ALD growth rate (Figure 2a–c). To investigate the cause of this discrepancy, elemental mapping was conducted using EDS, and the results are discussed in the following section.

As shown in Figure 2d, the SAED pattern of the ZnO sample exhibits discrete rings corresponding to the hexagonal wurtzite structure (ICDD card No. 36-1451). As the Al concentration increases, additional diffraction spots corresponding to Al_2_O_3_ become increasingly prominent (Figure 2e,f). These findings indicate that high Al doping promotes the formation of both polycrystalline ZnO and secondary Al_2_O_3_ (ICDD card No. 46-1212) phases within the film. Although Al_2_O_3_ has been used in previous studies as a carrier suppressor due to its strong Al–O bonds [34], its unintended formation in this study is undesirable, as it contradicts the intended enhancement of carrier concentration in ZnO-based thin films.

**Figure 3:**
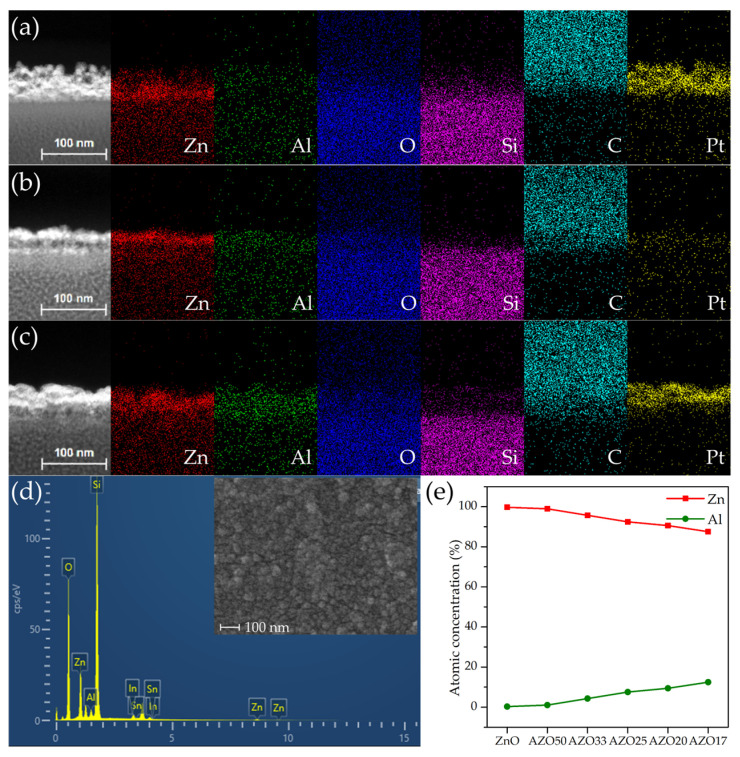
Elemental mapping results of cross-sectional HAADF TEM images for (**a**) ZnO, (**b**) AZO25 and (**c**) AZO17. (**d**) Surface EDS spectrum of the AZO17 film with an inset of the corresponding plane-view FE-SEM image. (**e**) Variation in the atomic ratios of Al and Zn calculated from EDS analysis with increasing number of Al_2_O_3_ ALD cycles.

To accurately determine the thickness of the deposited films and to investigate the variation in the Al-to-Zn ratio with increasing Al_2_O_3_ cycle numbers, EDS analysis was performed. As shown in Figure 3a–c, regions with strong Zn signals exhibited a thickness of approximately 20–30 nm. In contrast, other regions displayed a dominant Pt signal. To verify that Pt was not intrinsic to the device structure, an additional EDS analysis was performed on the uncoated surface of the AZO17 sample using FE-SEM, as shown in the inset of Figure 3d. No Pt signal was detected, supporting the conclusion that Pt observed in the TEM analysis originated from FIB-induced redeposition. Since Pt was not involved in the device fabrication process, the presence of Pt is attributed to re-deposition during TEM sample preparation using FIB milling. Such Pt contamination during FIB processing has also been reported in previous studies [35,36].

The deposited films did not exhibit uniform thickness, as evidenced by the EDS mapping results shown in Figure 3a–c, where Zn was unevenly distributed across the cross-sections in all samples. This non-uniformity is also apparent in the surface morphology of the AZO17 sample, as shown in the plane-view FE-SEM image in the inset of Figure 3d. This phenomenon is attributed to poor adhesion at the glass interface, which likely caused the initial growth to proceed in an island-like mode rather than forming a continuous film [37,38]. The surface roughness appears to have been further enhanced by the post-deposition annealing process, possibly due to grain growth and coalescence [39]. This morphological non-uniformity may lead to an increase in the specific surface area, thereby providing a greater number of active adsorption sites, which can enhance gas–solid interactions. Similar correlations between increased surface area and improved sensing performance have been widely reported in nanostructured metal oxide sensors [40,41].

The elemental mapping results confirm that Al, Zn, and O are homogeneously distributed throughout the thin films, indicating uniform incorporation of Al dopants into the ZnO matrix. To evaluate the relative doping trend, EDS analysis was performed exclusively on the deposited regions of all samples. As the number of Al_2_O_3_ cycles increased, the relative atomic ratio of Al increased, whereas that of Zn decreased, as presented in Figure 3e. The correlation confirms that adjusting the super-cycle composition enables modulation of the Al doping concentration in the ZnO.

### 3.2. XPS Analysis

To investigate the relative atomic composition of Zn and Al, and the chemical bonding states of O under varying Al doping concentrations, XPS analyses were conducted. All spectra were calibrated with reference to appropriate binding energy standards.

**Figure 4:**
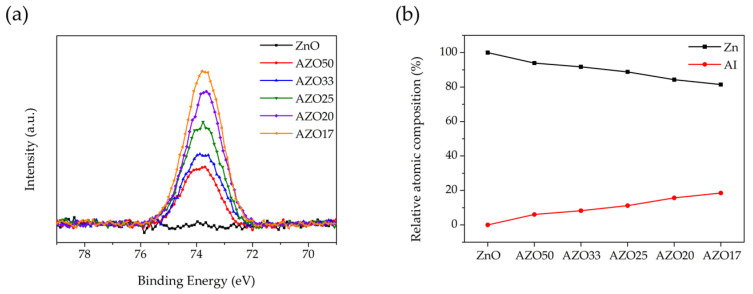
XPS spectra of (**a**) the Al 2p region for ZnO-based thin films and (**b**) relative atomic composition of Zn and Al obtained from XPS survey spectra with varying Al doping concentration.

As shown in Figure 4a, the XPS spectrum of ZnO exhibited no detectable peak near 74 eV [34], indicating the absence of Al in the undoped ZnO film. In contrast, the Al 2p peak intensity increased progressively with the number of Al_2_O_3_ cycles in the AZO films. The relative atomic composition of Zn and Al was calculated using the XPS survey spectra (Figure 4b). The peak areas of Zn and Al were first obtained and then normalized using their respective relative sensitivity factors to determine the Zn:Al ratio. An increase in the Al atomic percentage was observed with increasing Al_2_O_3_ cycle number, accompanied by a corresponding decrease in Zn content. This compositional trend is consistent with the results obtained from EDS measurements. However, the Al doping concentration determined by XPS was higher than that obtained from EDS analysis. As XPS is a surface-sensitive technique, the Al doping concentration in the AZO films was likely overestimated due to the presence of the Al_2_O_3_ cycle deposited at the topmost surface in the final step of the ALD process.

**Figure 5:**
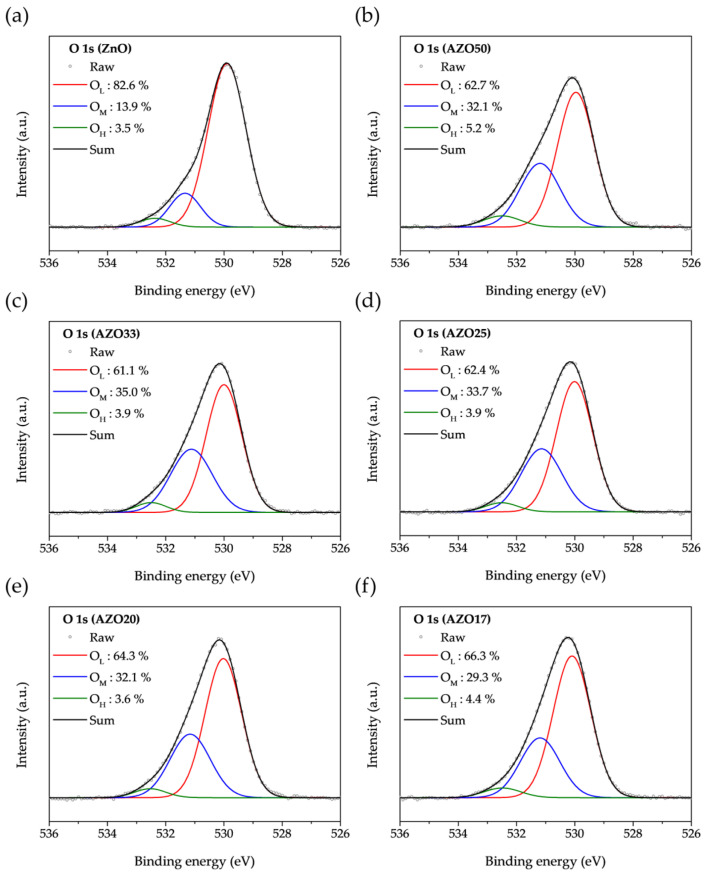
O 1s core level XPS spectra of (**a**) ZnO, (**b**) AZO50, (**c**) AZO33, (**d**) AZO25, (**e**) AZO20, and (**f**) AZO17.

The O 1s binding peaks of ZnO-based samples were fitted using Gaussian functions, as shown in Figure 5. The peaks located at 530.0 ± 0.1, 531.6 ± 0.1, and 532.6 ± 0.1 eV correspond to lattice oxygen (O_L_), oxygen vacancies (O_M_), and metal-hydroxyl bonding (O_H_), respectively [42,43]. Oxygen vacancies serve as the primary source of free electrons for n-type conductivity. Compared to ZnO, the AZO samples exhibited a larger O_M_ component, which is attributed to the strong reducing property of TMA, which induces the formation of oxygen vacancies in ZnO.

**Figure 6:**
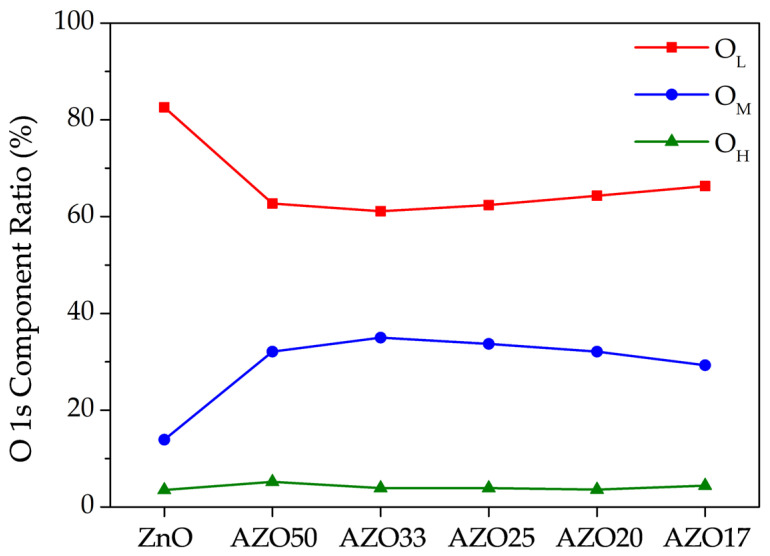
Relative ratios of the O_L_, O_M_, and O_H_ components extracted from the deconvoluted O 1s spectra shown in Figure 5.

To compare the variation in oxygen bonding states with increasing Al doping concentration, the O_L_, O_M_, and O_H_ components from Figure 5 are summarized in Figure 6. The highest ratio of oxygen vacancies was observed in AZO33, and it gradually decreased with further increases in the number of Al_2_O_3_ cycles. At low doping concentrations, Al primarily exists as Al^3+^ and either substitutes Zn^2+^ in the lattice or occupies interstitial sites, thereby increasing the electron concentration. However, at higher doping levels, Al tends to form Al_2_O_3_ [34]. Since Al_2_O_3_ involves stable bonding between metal and oxygen atoms, it is often regarded as a carrier suppressor. Furthermore, whereas Zn and O form a 1:1 stoichiometric ratio, Al and O tend to form a 1:1.5 ratio, which further suppresses the formation of oxygen vacancies.

As a result, a decreasing trend in the O_M_ component and a corresponding increase in the O_L_ component were observed at higher Al doping levels. The O_H_ component was consistently observed to be below 5% across all samples, likely due to the high annealing temperature.

### 3.3. Optical Properties

**Figure 7:**
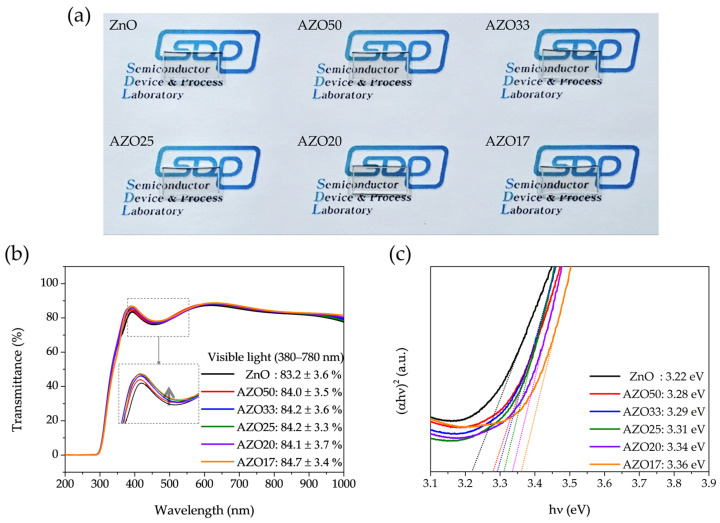
(**a**) Optical images, (**b**) transmittance spectra showing the average visible transmittance (380–780 nm), with a dashed box indicating the magnified region between 400–450 nm for clarity, and (**c**) Tauc plots, where the extrapolated linear region corresponds to the bandgap energy of ZnO and AZO thin films with varying Al concentrations.

Figure 7a presents the optical images of ZnO, AZO50, AZO33, AZO25, AZO20, and AZO17. The sample names are written under the samples, and their high optical transparency in the visible region allows the underlying image to remain clearly visible. A slight yellowish tint is also observed across the devices. Figure 7b presents the transmission spectra of pristine ZnO and AZO thin films, demonstrating that all ZnO-based films exhibit transmittance exceeding 80% in the visible range, with a slight reduction to approximately 75% in the 400–500 nm region. This partial decrease in blue-light transmittance accounts for the yellowish appearance observed in Figure 7a, consistent with previously reported optical characteristics of ITO-based films [44]. Additionally, in the 350–450 nm wavelength range, all AZO devices exhibited enhanced transmittance compared to undoped ZnO devices.

The absorption coefficient (*α*) of AZO films with varying Al concentrations was determined using Lambert’s law, as given in Equation (1):

*α* = 1/*t* ln(1/*T*) (1)

where *t* is the film thickness and *T* is the transmittance.

To determine the optical direct band gap (*E_g_*), Tauc’s relation was applied, as expressed in Equation (2):

(*αhν*)^2^ = *A*(*hν* − *E*_*g*_) (2)

where *h* is Planck’s constant, *ν* is the frequency of the light, and *A* is a constant. The *E*_*g*_ values of the thin films were obtained by extrapolating the linear region of the (*αhν*)^2^ vs. *hν* plot to the *hν*-axis, as shown in Figure 7c. As the Al doping concentration increased, the optical band gap widened from 3.22 eV to 3.36 eV.

Two mechanisms may account for this trend. The first is the Burstein–Moss effect [45,46], in which increased electron concentration fills the lower energy states of the conduction band, resulting in an apparent widening of the optical bandgap. The second is structural modification due to Al–O bonding and partial formation of Al_2_O_3_ phases, which can intrinsically alter the band structure of ZnO [34]. Both effects may contribute, depending on the doping level and local structural changes. Specifically, the Burstein–Moss effect dominates at low doping concentrations, where oxygen vacancies are abundant. However, at higher doping levels, vacancy suppression caused by strong Al–O bonding reduces the free carrier density, while the structural effect continues to increase, due to the incorporation of Al into the lattice. As a result, the combined influence of these two mechanisms leads to a non-linear increase in optical transmittance. Within the visible-light energy range (1.6–3.3 eV), this band gap increase enhances the optical transparency of the films. Overall, these results demonstrate a slight improvement in the optical properties due to Al doping.

### 3.4. Gas-Sensing Properties

#### 3.4.1. Gas-Sensing Mechanism

The NO_2_ sensing mechanism in ZnO involves surface interactions between adsorbed gas molecules and free carriers in the MOS. NO_2_ molecules extract electrons from the MOS during adsorption, thereby expanding the surface depletion region and increasing the electrical resistance. Sensing performance can be enhanced through the strategic introduction of oxygen vacancies, introduced through TMA exposure during the ALD process employed herein. Oxygen vacancies introduce shallow donor levels near the conduction band in n-type metal oxide semiconductors, thereby increasing electron density and facilitating carrier transport [27,29]. In addition, they promote surface charge transfer by providing active adsorption sites for NO_2_, which contributes to enhanced gas-sensing performance [27,29,30]. In our AZO films, these oxygen vacancy-mediated effects collectively contribute to the enhanced NO_2_ sensing performance, highlighting the effectiveness of controlled defect engineering via ALD.

In contrast, the response mechanism to reducing gases such as CO and NH_3_ involves surface reactions with chemisorbed oxygen species, through reactions like CO + O^−^ → CO_2_ + e^−^ and NH_3_ + O^−^ → N_2_ + H_2_O + e^−^ [5,47]. Although Al doping increases the concentration of oxygen vacancies and thereby promotes the formation of these chemisorbed species, slightly enhancing the response to reducing gases, its dominant effect is the increased free carrier concentration, which substantially amplifies the interaction with oxidizing gases such as NO_2_.

However, at high Al doping concentrations, the formation of electrically insulating Al_2_O_3_ phases suppresses the generation of oxygen vacancies, thereby diminishing the overall sensing response. It is worth noting that the concentration of oxygen vacancies is influenced not only by the reducing nature of TMA, but also by the extent of Al_2_O_3_ formation. In this study, the Al doping concentration was carefully optimized to balance these effects, resulting in improved NO_2_ sensing performance of the AZO films.

#### 3.4.2. Gas-Sensing Characteristics

**Figure 8:**
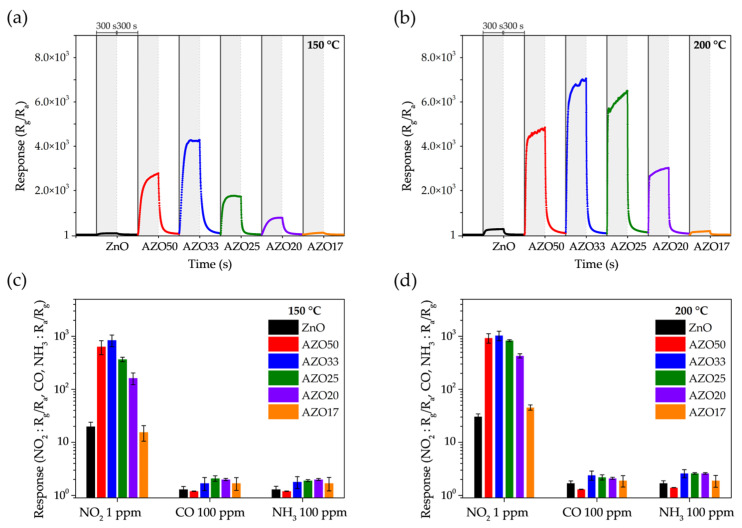
Dynamic gas response of ZnO-based sensors to 5 ppm NO_2_ at (**a**) 150 °C and (**b**) 200 °C. Selectivity of the sensors toward 1 ppm NO_2_, 100 ppm CO, and 100 ppm NH_3_ at (**c**) 150 °C and (**d**) 200 °C, with standard deviation error bars.

As shown in Figure 8a,b, the gas-sensing characteristics of the transparent sensors were evaluated upon exposure to 5 ppm NO_2_ at 150 °C and 200 °C. The baseline resistance, R_a_, and the corresponding response and recovery times are summarized in Table 1. The pristine ZnO exhibited responses of 64.6 and 255.2 at 150 °C and 200 °C, respectively. In contrast, AZO33 showed the highest response among all samples, reaching 4277.3 at 150 °C and 7058.7 at 200 °C, which are approximately 66.2 and 27.7 times higher than those of pristine ZnO. All AZO samples, except AZO17, exhibited enhanced NO_2_ sensitivity compared to un-doped ZnO. Furthermore, all samples demonstrated improved sensing performance as the operating temperature increased from 150 °C to 200 °C.

As shown in Table 1, the trends in sensitivity and baseline resistance with respect to Al doping concentration closely follow the variation in oxygen vacancy ratios observed in the XPS analysis. Oxygen vacancies act as primary electron donors in n-type MOSs, and all Al-doped samples exhibited lower R_a_ than pristine ZnO. Specifically, AZO33, which showed the highest oxygen vacancy concentration, exhibited the lowest baseline resistance. In addition, Al doping led to reduced response and recovery times compared to pristine ZnO, particularly at moderate doping levels, indicating enhanced reaction kinetics toward NO_2_ gas. However, when the Al concentration becomes excessively high—as in the case of AZO17—the formation of electrically insulating Al_2_O_3_ suppresses the generation of oxygen vacancies and hinders gas–MOSs interactions, ultimately resulting in deteriorated sensing performance.

Figure 8c,d present the gas selectivity of ZnO and AZO samples toward 1 ppm NO_2_, 100 ppm CO, and 100 ppm NH_3_ at operating temperatures of 150 °C and 200 °C. The error bars represent standard deviation from multiple measurements. CO is a candidate biomarker and is commonly co-emitted with NO_2_ in industrial settings. NH_3_ is a known biomarker for conditions such as renal dysfunction and Helicobacter pylori-related gastric infections, and may also be present alongside NO_2_ in medical contexts. Thus, achieving selectivity over these gases is critical for real-world NO_2_ sensing applications.

While all AZO samples exhibited significantly enhanced responses to NO_2_, their responses to CO and NH_3_ remained relatively low. At 150 °C, the responses to CO and NH_3_ increased only marginally—from 1.3 to a maximum of 2.1 (1.6-fold) and from 1.3 to a maximum of 2.0 (1.5-fold), respectively—compared to pristine ZnO. In contrast, the NO_2_ response of the AZO33 sample increased dramatically, by a factor of 42.4. As a result, the NO_2_ selectivity over CO and NH_3_ was significantly enhanced, reaching 32.6 and 30.3, respectively. A similar trend was observed at 200 °C, with NO_2_ selectivity over CO and NH_3_ reaching 24.0 and 22.2, respectively.

The response to reducing gases such as CO and NH_3_ is attributed to surface reactions with chemisorbed oxygen species, which modulate the carrier concentration in the sensing layer. Al doping in the ZnO lattice increases the concentration of oxygen vacancies, thereby promoting the formation of these chemisorbed species and slightly enhancing the response to reducing gases. However, the dominant effect of Al doping is the increased free carrier concentration, which substantially amplifies the interaction with oxidizing gases such as NO_2_. This explains the significant improvement in both NO_2_ sensitivity and gas selectivity observed in the AZO sensors.

**Figure 9:**
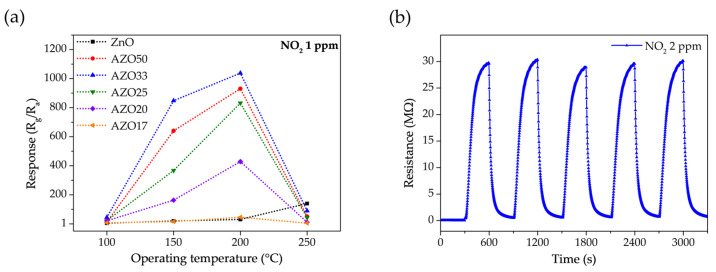
(**a**) Gas response of pristine ZnO and AZO sensors with various Al doping levels to 1 ppm NO_2_ as a function of operating temperature (100–250 °C). (**b**) Repeatability test of the AZO33 sensor under exposure to 2 ppm NO_2_ over five consecutive response/recovery cycles at 150 °C.

The gas response of ZnO-based sensors with various Al doping levels was investigated at temperatures ranging from 100 to 250 °C, as shown in Figure 9a. All AZO samples exhibited their maximum responses at 200 °C, whereas the pristine ZnO sensor reached its peak response at 250 °C. This indicates that Al doping not only enhances the gas-sensing performance, but also lowers the optimal operating temperature to 200 °C. Among the samples, AZO33 exhibited the highest response, with values of 44.9 at 100 °C, 847.9 at 150 °C, and 1037.9 at 200 °C.

Given that prolonged exposure to 1–3 ppm NO_2_ can lead to chronic respiratory conditions and that acute exposure above 5 ppm may cause severe pulmonary effects, reliable detection within this concentration range is critical [48]. Notably, even at the relatively low temperature of 100 °C, AZO33 demonstrated sufficient sensitivity to detect 1 ppm NO_2_, which corresponds to the occupational exposure limit, highlighting its practical applicability. This lower optimal operating temperature in Al-doped samples is advantageous for real-world applications in terms of power consumption.

**Figure 10:**
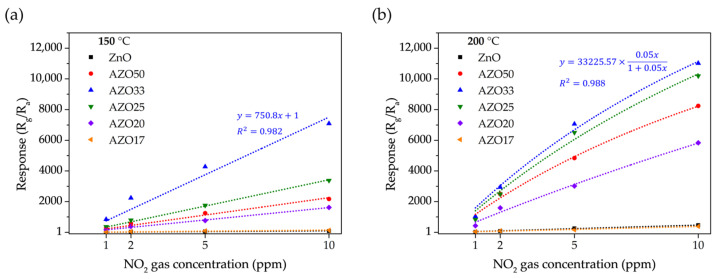
Dynamic response curves of the fabricated sensors upon exposure to NO_2_ concentrations ranging from 1 to 10 ppm at (**a**) 150 °C with linear fitting and (**b**) 200 °C with Langmuir model fitting.

Figure 9b presents the dynamic response of the AZO33 sensor to five consecutive exposure/recovery cycles under 2 ppm NO_2_ at 150 °C. The peak resistance values showed minimal variation, fluctuating within ±2.5% across all cycles. Moreover, the response and recovery kinetics remained consistent throughout the test, indicating reliable and reproducible sensing behavior under repeated NO_2_ exposure. These results demonstrate the high stability, repeatability, and reversibility of the AZO33 sensor. Gas response characteristics to various concentrations of NO_2_ were measured at 150 °C and 200 °C, as shown in Figure 10. At 150 °C, most samples exhibited a relatively linear increase in response with increasing NO_2_ concentration. AZO33, which exhibited the highest response at 150 °C, showed a fitted response of *y* = 750.8*x* + 1 with an R^2^ value of 0.983, indicating reliable linearity. In contrast, at 200 °C, several sensors, particularly those with higher sensitivity, displayed a reduced slope at higher concentrations. This saturation behavior can be explained by the depletion of available adsorption sites, especially in high-sensitivity sensors with thin-film structures. This behavior is consistent with a Langmuir-type isotherm, where the sensor response increases linearly at low gas concentrations but tends to saturate as the surface sites become fully occupied [49]. The AZO33 sensor at 200 °C, which exhibited the highest sensitivity, showed an excellent fit to the Langmuir adsorption model (*y* = 33,225.57 × 0.05*x*/(1 + 0.05*x*)) with an R^2^ value of 0.988, supporting the surface-site saturation behavior.

Although this study was limited to ppm-level measurements due to equipment constraints, the AZO33 sensor demonstrated a strong response even at 1 ppm, as shown in Figure 10. This suggests that it may be capable of detecting NO_2_ at sub-ppm or ppb levels, highlighting its potential for highly sensitive environmental monitoring applications.

Table 2 summarizes the NO_2_ sensing characteristics of various doped ZnO-based sensors in comparison with previously reported studies. To enable direct comparison with previous works, the response values were recalculated using the formula (R_g_ − R_a_)/R_a_ × 100. Using this metric, the AZO33 sample exhibited significant responses of 4390% at 100 °C and 84,690% at 150 °C. This performance surpasses that of comparable sensors in the literature, highlighting the impact of the material design strategy employed in this work. In particular, the combination of Al doping and oxygen vacancy engineering played a key role in enhancing gas-sensing behavior. These findings offer meaningful guidance for the advancement of transparent oxide sensors designed for efficient sensing at moderately low temperatures.

## 4. Conclusions

This study demonstrated an effective strategy to enhance the NO_2_ sensing performance of transparent ZnO-based gas sensors by engineering the carrier concentration. The concentration of oxygen vacancies, which act as the primary electron donors in n-type semiconductors, was intentionally increased using TMA, a highly reducing precursor. Cross-sectional TEM analysis revealed comparable film thicknesses between the pristine and highly Al-doped samples. SAED analysis showed the formation of polycrystalline Al_2_O_3_ phases at high doping concentrations, which coincided with a reduction in oxygen vacancy concentration, as confirmed by XPS. An initial increase in oxygen vacancies was observed at low doping levels, followed by a decline beyond the AZO33 sample, a trend that aligned with changes in baseline resistance. Furthermore, UV–Vis spectroscopy revealed a progressive widening of the optical bandgap with increasing Al content, accompanied by improved transmittance in the visible region.

The AZO33 sensor, which exhibited the highest oxygen vacancy concentration, demonstrated the most outstanding NO_2_ sensing performance. Compared to pristine ZnO, the AZO33 sensor showed approximately 66.2-fold higher sensitivity at 150 °C and 27.7-fold higher sensitivity at 200 °C. The selectivity toward NO_2_ over CO and NH_3_ increased 32.6-fold and 30.3-fold at 150 °C, and 24.0-fold and 22.2-fold at 200 °C, respectively. In addition, both the response and recovery times were significantly improved. The optimal operating temperature was reduced by 50 °C, and reliable detection of 1 ppm NO_2_ was achieved even at 100 °C. These improvements were achieved by systematically tuning the Al doping concentration, which effectively optimized the gas-sensing properties of AZO. These results highlight the potential of AZO sensors for low-power and practical environmental monitoring applications. To further expand their applicability, future work will explore low-temperature fabrication and operation strategies suitable for flexible platforms, and investigate humidity effects and long-term stability, which are critical for real-world use.

## Figures and Tables

**Table 1:** Gas-sensing parameters of ZnO and AZO sensors at 150 °C and 200 °C, corresponding to the dynamic responses in Figure 8a,b.

Temperature (°C)	Sample	R_a_ (Ω)	Response	Response Time (s)	Recovery Time (s)
150	ZnO	2.0 × 10^6^	64.6	106	252
	AZO50	1.0 × 10^5^	2776.8	148	72
	AZO33	8.8 × 10^4^	4277.3	92	90
	AZO25	1.6 × 10^5^	1758.9	70	40
	AZO20	3.1 × 10^5^	767.3	232	104
	AZO17	4.6 × 10^5^	91.5	230	158
200	ZnO	1.1 × 10^5^	255.2	98	52
	AZO50	2.8 × 10^4^	4841.2	72	28
	AZO33	2.6 × 10^4^	7058.7	72	34
	AZO25	3.6 × 10^4^	6502.7	94	28
	AZO20	3.8 × 10^4^	3020.0	54	30
	AZO17	6.1 × 10^4^	165.0	207	44

**Table 2:** Comparison of NO_2_ sensing responses of AZO sensors in this study with previously reported ZnO-based sensors. (Data adapted from References [31,32,33,49,50].)

Sample	Concentration (ppm)	Operating Temperature (°C)	Response Equation	Response	Reference
Al-doped ZnO nanorod	5	175	(R_g_ − R_a_)/R_a_ × 100	85%	[31]
Al-doped ZnO nanostructure	1	240	R_g_/R_a_	103.98	[32]
Al-doped ZnO nanocomposite	5	200	R_g_/R_a_	13.27	[33]
Fe-doped ZnO thin film	10	200	R_g_/R_a_	2.5	[50]
TeO_2_-doped ZnO nanostructure	1	100	(R_g_ − R_a_)/R_a_ × 100	80%	[51]
Al-doped ZnO thin film (AZO33)	1	100	R_g_/R_a_	44.9	This work
	1	150	R_g_/R_a_	847.9	

## Data Availability

The datasets generated and/or analyzed during the current study are available from the corresponding author on reasonable request.

## Abbreviations

The following abbreviations are used in this manuscript:

IoT	Internet of Things
MOS	Metal oxide semiconductor
ALD	Atomic layer deposition
TMA	Trimethylaluminum
AZO	Al-doped ZnO
DEZ	Diethylzinc
TEM	Transmission electron microscopy
SAED	Selected area electron diffraction
EDS	Energy-dispersive X-ray spectroscopy
FE-SEM	Field-emission scanning electron microscope
XPS	X-ray photoelectron spectroscopy
UV-Vis	Ultraviolet–visible spectroscopy
FIB	Focused ion beam

## References

[1] Nomura K., Ohta H., Takagi A., Kamiya T., Hirano M., Hosono H. (2004). Room-temperature fabrication of transparent flexible thin-film transistors using amorphous oxide semiconductors. Nature.

[2] Lee J.-Y., Connor S.T., Cui Y., Peumans P. (2008). Solution-Processed Metal Nanowire Mesh Transparent Electrodes. Nano Lett..

[3] Ishikawa F.N., Chang H.-K., Ryu K., Chen P.-C., Badmaev A., Gomez De Arco L., Shen G., Zhou C. (2009). Transparent Electronics Based on Transfer Printed Aligned Carbon Nanotubes on Rigid and Flexible Substrates. ACS Nano.

[4] Zong B., Wu S., Yang Y., Li Q., Tao T., Mao S. (2024). Smart Gas Sensors: Recent Developments and Future Prospective. Nanomicro Lett..

[5] Hakeem Anwer A., Saadaoui M., Mohamed A.T., Ahmad N., Benamor A. (2024). State-of-the-Art advances and challenges in wearable gas sensors for emerging applications: Innovations and future prospects. Chem. Eng. J..

[6] Bandodkar A.J., Jeerapan I., Wang J. (2016). Wearable Chemical Sensors: Present Challenges and Future Prospects. ACS Sens..

[7] Tai H., Wang S., Duan Z., Jiang Y. (2020). Evolution of breath analysis based on humidity and gas sensors: Potential and challenges. Sens. Actuators B Chem..

[8] Kim S.J., Choi S.J., Jang J.S., Cho H.J., Kim I.D. (2017). Innovative Nanosensor for Disease Diagnosis. Acc. Chem. Res..

[9] Zheng Z.Q., Yao J.D., Wang B., Yang G.W. (2015). Light-controlling, flexible and transparent ethanol gas sensor based on ZnO nanoparticles for wearable devices. Sci. Rep..

[10] Kim Y.H., Kim S.J., Kim Y.-J., Shim Y.-S., Kim S.Y., Hong B.H., Jang H.W. (2015). Self-Activated Transparent All-Graphene Gas Sensor with Endurance to Humidity and Mechanical Bending. ACS Nano.

[11] Loghin F.C., Falco A., Salmeron J.F., Lugli P., Abdellah A., Rivadeneyra A. (2019). Fully Transparent Gas Sensor Based on Carbon Nanotubes. Sensors.

[12] Umar A., Akbar S., Kumar R., Amu-Darko J.N.O., Hussain S., Ibrahim A.A., Alhamami M.A., Almehbad N., Almas T., Seliem A.F. (2024). Ce-doped ZnO nanostructures: A promising platform for NO_2_ gas sensing. Chemosphere.

[13] Freddi S., Rodriguez Gonzalez M.C., Casotto A., Sangaletti L., De Feyter S. (2023). Machine-Learning-Aided NO_2_ Discrimination with an Array of Graphene Chemiresistors Covalently Functionalized by Diazonium Chemistry. Chemistry.

[14] Mokrushin A.S., Gorban Y.M., Averin A.A., Gorobtsov P.Y., Simonenko N.P., Lebedinskii Y.Y., Simonenko E.P., Kuznetsov N.T. (2023). Obtaining of ZnO/Fe_2_O_3_ Thin Nanostructured Films by AACVD for Detection of ppb-Concentrations of NO_2_ as a Biomarker of Lung Infections. Biosensors.

[15] Brophy R.E., Junker B., Fakhri E.A., Árnason H.Ö., Svavarsson H.G., Weimar U., Bârsan N., Manolescu A. (2024). Ultra Responsive NO_2_ silicon nanowires gas sensor. Sens. Actuators B Chem..

[16] Liu L., Wang Y., Liu Y., Wang S., Li T., Feng S., Qin S., Zhang T. (2022). Heteronanostructural metal oxide-based gas microsensors. Microsyst. Nanoeng..

[17] Sun Y.F., Liu S.B., Meng F.L., Liu J.Y., Jin Z., Kong L.T., Liu J.H. (2012). Metal oxide nanostructures and their gas sensing properties: A review. Sensors.

[18] Li S., Zhang M., Wang H. (2021). Simulation of gas sensing mechanism of porous metal oxide semiconductor sensor based on finite element analysis. Sci. Rep..

[19] Kohlmann N., Hansen L., Lupan C., Schurmann U., Reimers A., Schutt F., Adelung R., Kersten H., Kienle L. (2021). Fabrication of ZnO Nanobrushes by H_2_-C_2_H_2_ Plasma Etching for H_2_ Sensing Applications. ACS Appl. Mater. Interfaces.

[20] Kwak C.-H., Woo H.-S., Abdel-Hady F., Wazzan A.A., Lee J.-H. (2016). Vapor-phase growth of urchin-like Mg-doped ZnO nanowire networks and their application to highly sensitive and selective detection of ethanol. Sens. Actuator B Chem..

[21] Woo H.S., Na C.W., Lee J.H. (2016). Design of Highly Selective Gas Sensors via Physicochemical Modification of Oxide Nanowires: Overview. Sensors.

[22] Bae J., Jeon H. (2025). Characteristics of aluminum-doped SnO_2_ in various positions using super-cycle ALD. Nanotechnology.

[23] Yoo R., Cho S., Song M.-J., Lee W. (2015). Highly sensitive gas sensor based on Al-doped ZnO nanoparticles for detection of dimethyl methylphosphonate as a chemical warfare agent simulant. Sens. Actuators B Chem..

[24] Seok T.J., Liu Y., Choi J.H., Kim H.J., Kim D.H., Kim S.M., Jang J.H., Cho D.-Y., Lee S.W., Park T.J. (2020). In Situ Observation of Two-Dimensional Electron Gas Creation at the Interface of an Atomic Layer-Deposited Al_2_O_3_/TiO_2_ Thin-Film Heterostructure. Chem. Mater..

[25] Baek D., Lee S.-H., Bak S.-Y., Jang H., Lee J., Yi M. (2024). Control of Threshold Voltage in ZnO/Al_2_O_3_ Thin-Film Transistors through Al_2_O_3_ Growth Temperature. Electronics.

[26] Eom H., Bae W., Sung J.Y., Choi J.H., Dae K.S., Jang J.H., Park T.J., Lee S.W., Shong B. (2024). Formation of oxygen vacancy at surfaces of ZnO by trimethylaluminum. APL Mater..

[27] Zhang C., Liu G., Geng X., Wu K., Debliquy M. (2020). Metal oxide semiconductors with highly concentrated oxygen vacancies for gas sensing materials: A review. Sens. Actuators A Phys..

[28] Fan Y., Song L., Wang W., Fan H. (2025). Nano-Micro Structure of Metal Oxide Semiconductors for Triethylamine Sensors: ZnO and In_2_O_3_. Nanomaterials.

[29] Gao R., Wang S., Xu Y., Zhang X., Gao J., Zheng M., Zhou X., Huo L. (2025). Dual-defect enhanced NO_2_ sensing performance at low power consumption of ZnO-ZnTe core-shell nanorods via one-step controllable assembly. J. Alloys Compd..

[30] Li P., Fan H., Cai Y., Xu M., Long C., Li M., Lei S., Zou X. (2014). Phase transformation (cubic to rhombohedral): The effect on the NO_2_ sensing performance of Zn-doped flower-like In_2_O_3_ structures. RSC Adv..

[31] Patil V.L., Dalavi D.S., Dhavale S.B., Tarwal N.L., Vanalakar S.A., Kalekar A.S., Kim J.H., Patil P.S. (2022). NO_2_ gas sensing properties of chemically grown Al doped ZnO nanorods. Sens. Actuators A Phys..

[32] Zhang Y.-H., Li Y.-L., Gong F.-L., Xie K.-F., Liu M., Zhang H.-L., Fang S.-M. (2020). Al doped narcissus-like ZnO for enhanced NO_2_ sensing performance: An experimental and DFT investigation. Sens. Actuators B Chem..

[33] Park S., Eom T.-y., Jeong R.-H., Lee H.-J., Boo J.-H. (2024). Synthesis and characterization of Al-doped ZnO/CdO heterostructured nanocomposites for enhancing NO_2_ gas sensing performance. Appl. Surf. Sci..

[34] Maeng W.J., Lee J.-w., Lee J.H., Chung K.-B., Park J.-S. (2011). Studies on optical, structural and electrical properties of atomic layer deposited Al-doped ZnO thin films with various Al concentrations and deposition temperatures. J. Phys. D Appl. Phys..

[35] Lee S.B., Park J., van Aken P.A. (2016). Formation of Pt–Zn alloy nanoparticles by electron-beam irradiation of wurtzite ZnO in the TEM. Nanoscale Res. Lett..

[36] Blanchet M.D., Matthews B.E., Spurgeon S.R., Heald S.M., Isaacs-Smith T., Comes R.B. (2023). Jahn–Teller-driven phase segregation in Mn_x_Co_3− x_O_4_ spinel thin films. J. Vac. Sci. Technol. A.

[37] Yen C.Y., Jian S.R., Chen G.J., Lin C.M., Lee H.Y., Ke W.C., Liao Y.Y., Yang P.F., Wang C.T., Lai Y.S. (2011). Influence of annealing temperature on the structural, optical and mechanical properties of ALD-derived ZnO thin films. Appl. Surf. Sci..

[38] Makino H., Kishimoto S., Yamada T., Miyake A., Yamamoto N., Yamamoto T. (2008). Effects of surface pretreatment on growth of ZnO on glass substrate. Phys. Status Solidi.

[39] Moeini B., Avval T.G., Brongersma H.H., Prusa S., Babik P., Vanickova E., Strohmeier B.R., Bell D.S., Eggett D., George S.M. (2023). Area-Selective Atomic Layer Deposition of ZnO on Si\SiO_2_ Modified with Tris(dimethylamino)methylsilane. Materials.

[40] Guan W., Tang N., He K., Hu X., Li M., Li K. (2020). Gas-Sensing Performances of Metal Oxide Nanostructures for Detecting Dissolved Gases: A Mini Review. Front. Chem..

[41] Lee J.-H. (2009). Gas sensors using hierarchical and hollow oxide nanostructures: Overview. Sens. Actuator B Chem..

[42] Hsieh P.T., Chen Y.C., Kao K.S., Wang C.M. (2007). Luminescence mechanism of ZnO thin film investigated by XPS measurement. Appl. Phys. A Mater. Sci. Process..

[43] Chen M., Wang X., Yu Y., Pei Z., Bai X., Sun C., Huang R., Wen L. (2000). X-ray photoelectron spectroscopy and auger electron spectroscopy studies of Al-doped ZnO films. Appl. Surf. Sci..

[44] DeAngelis A.D., Rougier A., Manaud J.-P., Labrugère C., Miller E.L., Gaillard N. (2014). Temperature-resistant high-infrared transmittance indium molybdenum oxide thin films as an intermediate window layer for multi-junction photovoltaics. Sol. Energy Mater. Sol. Cells.

[45] Ahmed G., Mohamed W.S., Hasaneen M.F., Ali H.M., Ibrahim E.M.M. (2023). Optical, structural, electrical and photocatalytic properties of aluminum doped zinc oxide nanostructures. Opt. Mater..

[46] Mamat M.H., Sahdan M.Z., Khusaimi Z., Ahmed A.Z., Abdullah S., Rusop M. (2010). Influence of doping concentrations on the aluminum doped zinc oxide thin films properties for ultraviolet photoconductive sensor applications. Opt. Mater..

[47] Fitriana F., Septiani N.L.W., Irzaman I., Ferdiansjah F., Fahmi M.Z., Adhika D.R., Suyatman S., Nugraha N., Yuliarto B. (2019). Preparation of (002)-oriented ZnO for CO gas sensor. Mater. Res. Express.

[48] Li K., Luo Y., Liu B., Gao L., Duan G. (2019). High-performance NO_2_-gas sensing of ultrasmall ZnFe_2_O_4_ nanoparticles based on surface charge transfer. J. Mater. Chem. A.

[49] Kumar R.R., Murugesan T., Dash A., Hsu C.-H., Gupta S., Manikandan A., Anbalagan A.K., Lee C.-H., Tai N.-H., Chueh Y.-L. (2021). Ultrasensitive and light-activated NO_2_ gas sensor based on networked MoS_2_/ZnO nanohybrid with adsorption/desorption kinetics study. Appl. Surf. Sci..

[50] Hiremath M.V., Momin N., Kangralkar M.V., Manjanna J., Hegde B.G., Byalollikar D.G. (2024). Synthesis and characterization of Fe-doped ZnO films for enhanced NO_2_ gas-sensing applications. J. Korean Phys. Soc..

[51] Nagarjuna Y., Hsiao Y.-J. (2024). TeO_2_ doped ZnO nanostructure for the enhanced NO_2_ gas sensing on MEMS sensor device. Sens. Actuators B Chem..

